# An examination of introgression and incomplete lineage sorting among three closely related species of chocolate‐dipped damselfish (genus: *Chromis*)

**DOI:** 10.1002/ece3.5142

**Published:** 2019-04-19

**Authors:** Song He, Vanessa Robitzch, Jean‐Paul A. Hobbs, Michael J. Travers, Diego Lozano‐Cortés, Michael L. Berumen, Joseph D. DiBattista

**Affiliations:** ^1^ Division of Biological and Environmental Science and Engineering, Red Sea Research Center King Abdullah University of Science and Technology Thuwal Saudi Arabia; ^2^ School of Molecular and Life Sciences Curtin University Perth Western Australia Australia; ^3^ Australian Institute of Marine Science Indian Oceans Marine Research Centre Crawley Western Australia Australia; ^4^ Australian Museum Research Institute Australian Museum Sydney New South Wales Australia

**Keywords:** *Chromis*, connectivity, coral reef fishes, hybridization, Indo‐West Pacific, marine biogeography, microsatellite, mitochondrial DNA, Pomacentridae, population genetics

## Abstract

**Aim:**

To determine the impact of ecological and environmental histories on the evolution of coral reef damselfishes at two adjacent marine biogeographic suture zones.

**Location:**

Indo‐West Pacific, notably including two suture zones: Socotra and Christmas and Cocos/Keeling Islands.

**Taxon:**

*Chromis dimidiata*, *Chromis margaritifer*, and *Chromis fieldi*.

**Methods:**

We utilized a combination of nuclear and mitochondrial genetic markers in addition to visual abundance survey data of these fishes.

**Results:**

Despite genetic patterns consistent with incomplete lineage sorting and relatively low genetic differentiation among the three studied *Chromis* species, there is evidence of hybridization between *C. margaritifer* and *C. fieldi* at Christmas Island based on molecular and visual identification. Introgression appears to be spreading westwards to other *C. fieldi* populations based on COI haplotype comparison. Moreover, the genetic distance between *C. margaritifer* and *C. fieldi* suggests that Pleistocene sea‐level fluctuations may have contributed to allopatric divergence and secondary contact between these two closely related species.

**Main conclusions:**

Our study highlights that evolutionary processes in coral reef fishes operate differently between suture zones, possibly due to different ecological and environmental predispositions regulating secondary contact of sister species. While secondary contact likely led to hybridization and introgression at Christmas and Cocos/Keeling Islands, none of those processes seem present at Socotra for the chocolate‐dipped damselfish. This difference is likely due to an environmental barrier caused by hydrodynamic regimes in the Gulf of Aden.

## 1 INTRODUCTION

In the terrestrial environment, borders of biogeographical provinces represent areas where regional biotas come into contact (termed suture zones; Hewitt, 2000; Remington, 1968). Secondary contact between closely related species (i.e., sister species) leads to high levels of hybridization at these borders (Hewitt, 2000; Hobbs, Herwerden, Pratchett, & Allen, 2013). Suture zones are phylogenetically important as hybridization can have significant consequences to speciation processes (Abbott, Hegarty, Hiscock, & Brennan, 2010; Mallet, 2007). Hybrids may (a) be unfit and favor reproductive isolation between species (Via, 2009; Wu, 2001), (b) be fit and facilitate recurring introgression and reverse speciation (Harrison et al., 2017; Kleindorfer et al., 2014), or (c) even generate new species and radiation events (Mallet, 2007; Seehausen, 2004).

Despite a largely continuous expanse of ocean from the Red Sea to the East Pacific, marine research has identified at least six distinct marine biogeographical provinces in the tropical Indo‐West Pacific, which are consistently supported by genetic evidence (Bowen et al., 2016; Briggs & Bowen, 2012; Spalding et al., 2007). Although hybridization was traditionally deemed to be rare in the tropical marine environment (Hubbs, 1955), it is now considered common and appears to be concentrated at biogeographical borders (DiBattista et al., 2015; Hobbs & Allen, 2014; Hobbs, Frisch, Allen, & Herwerden, 2009; Montanari, Hobbs, Pratchett, & Herwerden, 2016). The Indian Ocean contains two recognized marine suture zones: (a) Christmas and Cocos/Keeling Islands in the eastern Indian Ocean (Hobbs & Allen, 2014), and (b) the Socotra Archipelago in the western Indian Ocean (DiBattista et al., 2015). These suture zones represent the junction of Indian Ocean fauna with Pacific Ocean and Red Sea fauna, respectively. The interaction between regional faunas provides the ideal opportunity to determine how hybridization and introgression at biogeographical borders affects phylogeography, phylogeny, and evolution of tropical marine organisms.

Hybridization at these marine suture zones was initially detected through field observations of hybrids with intermediate phenotypes and was later confirmed with genetic analyses. The intermediate coloration pattern of many reef fish hybrids makes them stand out among their parent species (DiBattista et al., 2015; Hobbs & Allen, 2014). Although intermediate coloration might not be conclusive evidence for hybridization, it has proven to be a reliable indicator (DiBattista et al., 2015). For example, hybridization between eight pairs of reef fish species in the Cocos–Christmas suture zone was first identified based on intermediate coloration and was later supported by observations of heterospecific breeding pairs, heterospecific social groups, and genetic data (DiBattista et al., 2012; Hobbs et al., 2009; Marie, Herwerden, Choat, & Hobbs, 2007; Montanari, Hobbs, Pratchett, Bay, & Herwerden, 2014; Payet et al., 2016; Yaakub, Bellwood, & Herwerden, 2007; Yaakub, Bellwood, Herwerden, & Walsh, 2006). In some cases, hybridization is not detected using coloration because hybridization occurred in distant past (Koblmüller, Egger, Sturmbauer, & Sefc, 2010; Kuriiwa, Hanzawa, Yoshino, Kimura, & Nishida, 2007), or hybrids are rare and/or because backcrossed individuals look like the parent species (Harrison et al., 2017). Thus, genetic markers are useful for revealing cryptic and historical cases of hybridization in our oceans.

Pomacentridae are one of the most speciose families of coral reef fishes (approx. 385 recognized species). Within this family, there is evidence of hybridization at biogeographical borders between species (Coleman et al., 2014; Harrison et al., 2017) and among different morphs/phenotypes within a species (van Herwerden & Doherty, 2006). While such secondary contact and hybridization has led to significant genomic introgression (e.g.,* Abudefduf* species) and may eventually lead to the loss of former endemic species (Coleman et al., 2014); in other damselfishes, hybridization could potentially be the main driver of diversification (e.g., anemonefishes; Litsios and Salamin (2014)). Determining how hybridization increases or decreases diversity in species‐rich families is essential to understanding how such richness evolved and is maintained.

*Chromis* is the most speciose genus within the family Pomacentridae and contains almost 100 described species to date (Eschmeyer, Fricke, Fong, & Polack, 2010; Randall & DiBattista, 2013). *Chromis dimidiata* (Klunzinger, 1871) was believed to be distributed from the Red Sea across the Indian Ocean to the coral triangle in the West Pacific. Since its original description, *C. dimidiata* has been split into three species. *Chromis margaritifer*, first described as a subspecies of *C. dimidiata* by Fowler (1946) due to different patterns of coloration, is now considered a valid species that is distributed from the West Pacific Ocean to Christmas Island in the eastern Indian Ocean. *Chromis dimidiata* populations in the central and western Indian Ocean were identified as a new species, *Chromis fieldi* (Randall & DiBattista, 2013) based on morphological and genetic separation. Hence, the distributional range of *C. dimidiata* is currently restricted to the Red Sea.

The range of these three closely related *Chromis* spp. covers at least two recognized marine suture zones for reef fish from the Red Sea through to the West Pacific Ocean: Christmas and Cocos/Keeling Islands (Hobbs & Allen, 2014) and the Socotra Archipelago (DiBattista et al., 2015). Putative hybrids between *C. fieldi* and *C. margaritifer* have been reported at the Cocos–Christmas suture zone Hobbs and Allen (2014), supported by heterospecific social groups and intermediate coloration; however, this has not yet been confirmed genetically. In contrast, individuals with intermediate coloration were not observed during field surveys at Socotra, but high rates of hybridization among coral reef fishes at this location (DiBattista et al., 2015) and lack of genetic identification of the region's specimen warrant a closer examination of its chocolate‐dipped damselfish. To further explore evolutionary relationships and confirm potential hybridization among these three *Chromis* spp., a thorough genetic investigation was conducted at several locations throughout their collective range by utilizing a combination of nuclear and mitochondrial markers, which included publicly available genetic sequences and phenotypic information.

## 2 MATERIALS AND METHODS

### 2.1 Sample collection and underwater visual surveys

**Figure 1:**
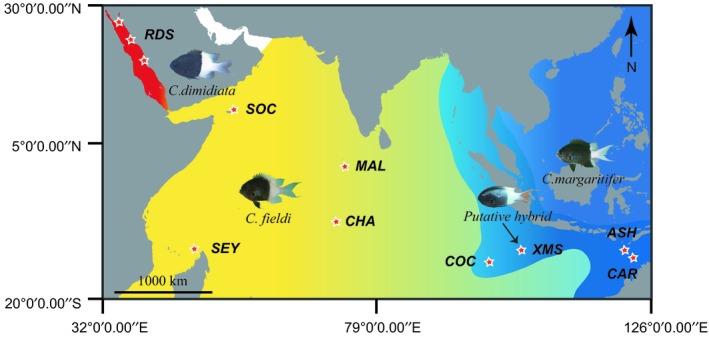
Map of sampling locations and putative geographic distribution ranges of *Chromis dimidiata* (red), *C. fieldi* (yellow), and *C. margaritifer* (blue). The shaded area between blue and yellow ranges indicates potential overlapping regions between the species *C. margaritifer* and *C. fieldi*. Collection sites are indicated by red stars on the map and locations are given code names as follows: ASH: Ashmore Reef; CAR: Cartier Reef; CHA: Chagos; COC: Cocos/Keeling Islands; MAL: Maldives; RDS: Red Sea; SEY: Seychelles; SOC: Socotra; XMS: Christmas Island. Putative hybrids were collected from XMS

Samples of *Chromis dimidiata*, *C. fieldi*, and *C. margaritifer* were collected between 2010 and 2014 across a broad geographical range (location abbreviations: RDS: Red Sea; SOC: Socotra Archipelago, Yemen; SEY: Seychelles; MAL: Maldives; CHA: Chagos; XMS: Christmas Island, Australia; COC: Cocos/Keeling Islands, Australia; ASH: Ashmore Reef, Australia; CAR: Cartier Reef, Australia) and identified in the field according to morphology and coloration pattern (Figure 1).

Specimens were preserved in saturated salt‐DMSO solution prior to DNA extraction. Sample size and collection locality of each species are indicated in Table 1. Four putative hybrids were identified by their intermediate body color and collected at sites where their putative parental species overlap (Figure 1). Underwater visual surveys were conducted to estimate species abundances based on three replicates of 50 m by 1 m transects at 5 m depth, at eight sites in Cocos/Keeling and seven sites in Christmas Island; at these sites *C. fieldi* and *C. margaritifer* overlap. Similar visual surveys were conducted at seven sampling sites on the north‐eastern coast of the main island of Socotra (also see DiBattista et al., 2015).

**Table 1:** Sample size and molecular diversity indices for *Chromis dimidiata*, *C. fieldi*, and *C. margaritifer* based on mitochondrial DNA cytochrome C oxidase subunit 1 (COI) sequences

Collection locality	*N*	*H_N_*	*τ*	Haplotype diversity (*h* ± *SD*)	Nucleotide diversity (*π* ± *SD*)	Fu's *F_S_*
*Chromis dimidiata*						
Red Sea, Saudi Arabia (RDS)	32 (26)	14	1.23	0.75 ± 0.08	0.0019 ± 0.0014	**−13.44**
*Chromis fieldi*						
Chagos Archipelago (CHA)	11 (11)	5	1.64	0.82 ± 0.08	0.0024 ± 0.0018	**−**1.27
Christmas Island (XMA)	2	n/a	n/a	n/a	n/a	n/a
Republic of Maldives (MAL)	6	3	1.57	0.60 ± 0.22	0.0016 ± 0.0015	1.56
Republic of Seychelles (SEY)	2	n/a	n/a	n/a	n/a	n/a
Socotra, Yemen (SOC)	8 (8)	2	0.83	0.53 ± 0.05	0.0009 ± 0.0009	1.36
All samples	29 (19)	6	1.09	0.67 ± 0.06	0.0016 ± 0.0013	**−**1.86
*Chromis margaritifer*						
Ashmore Reef, Aus. (ASH)	48 (49)	9	3.0	0.31 ± 0.09	0.0008 ± 0.0008	**−8.71**
Cartier Reef, Aus. (CAR)	23 (23)	5	2.98	0.32 ± 0.12	0.0006 ± 0.0007	**−3.90**
Christmas Island, Aus. (XMA)	83 (58)	17	0.49	0.39 ± 0.07	0.0008 ± 0.0008	**−24.58**
Cocos/Keeling Islands, Aus. (COC)	14 (14)	7	1.08	0.69 ± 0.14	0.0015 ± 0.0013	**−5.14**
All samples	168 (144)	29	0.56	0.38 ± 0.05	0.0008 ± 0.0008	n/a

Notes

Populations with *N* < 6 were not individually assessed but included in overall species calculations. Number of samples from each population included in microsatellites analysis is given in parentheses. Numbers in bold are significant, *p* < 0.02 as per Fu (1997).

*H_N_*: number of haplotypes; *τ*: population expansion parameter.

### 2.2 DNA extraction and sequencing

DNA was extracted from fish tissue using the “HotSHOT” protocol (Meeker, Hutchinson, Ho, & Trede, 2007) and samples were stored at −20°C. Mitochondrial DNA (mtDNA) fragments of the cytochrome c oxidase subunit one (COI) gene and nuclear DNA fragments of the recombination‐activating gene 2 (RAG2) were amplified using the primers FishF2 and FishR2 (Ward, Zemlak, Innes, Last, & Hebert, 2005) and the modified primers of (DiBattista et al., 2012), respectively. Polymerase chain reaction (PCR) mixes contained BioMix (BioMix Red; Bioline Ltd., London, UK), 0.26 μM of each primer, and 5–50 ng template DNA in 15 μl total volume. PCR reactions used the following cycling parameters: initial denaturing step at 95°C for 3 min, then 35 cycles of amplification (30 s at 94°C, 60 s at 50°C, and 60 s at 72°C), followed by a final extension at 72°C for 10 min.

All successfully amplified PCR products were cleaned by incubating with exonuclease I and shrimp alkaline phosphatase (ExoSAP; USB, Cleveland, OH, USA) at 37°C for 60 min, followed by 15 min at 85°C. Final products were sequenced in the forward (and reverse, for RAG2) direction with fluorescently labeled dye terminators following the manufacturer's protocols (BigDye version 3.1, Applied Biosystems Inc., Foster City, CA, USA) and on an ABI 3130XL Genetic Analyzer (Applied Biosystems).

Sequences were aligned, edited, and trimmed to same length using Geneious Pro *vers*. 4.8.4 (Drummond et al., 2009) and subsequently uploaded to GenBank (accession numbers: MH287769–MH287999). COI fragments from each species were queried using the BLAST tool on GenBank; all displayed 99% to 100% similarity to existing voucher sequences from each species (accession numbers: KF929750, JF493174–JF493176, JF434877–JF434880, JF434883, JF434885, JF434886, FJ583158–FJ583162). The listed voucher sequences were included in analyses, as indicated, for comparison. Details on the methodology for sequence analyses are in Supporting Information Appendix S1.

### 2.3 Microsatellite analysis

For a random subset of samples from all three species (Table 1, *N* in parentheses), nuclear microsatellite fragments were amplified using nine fluorescent labeled primer sets (Cm_A119, Cm_B007, Cm_B117, Cm_A110, Cm_A115, Cm_D006, Cm_A011, Cm_B102, and Cm_D114) developed by Underwood (2009) using following PCR conditions: initial denaturation step at 95°C for 15 min, then 25 cycles of amplification (40 s at 94°C, 40 s at 56°C, and 30 s at 72°C), followed by a final extension at 72°C for 10 min. PCR fragment length and quality were checked with the QIAxcel DNA High‐Resolution Kit (Qiagen, Hilden, Germany). Fragment lengths were estimated on a Sanger ABI 3730XL (Applied Biosystems). ABI files were imported into Geneious *vers*. 7.0.6 (Drummond et al., 2009), where alleles were scored by three independent researchers to confirm genotypes.

From all scored genotypes, those from loci CmA110 had almost nil variation across all the samples. Furthermore, three loci (CmB007, CmA110, and CmD114) were discarded due to low amplification success in >90% of the samples, and loci CmA115 was also discarded due to high null allele frequency at several sampled locations. The remaining five loci (CmA119, CmB117, CmD006, CmA011, and CmB102) met Hardy–Weinberg Equilibrium (HWE) assumptions, and did not show signs of linkage disequilibrium. Moreover, five out of 189 samples had data from more than one locus missing; those samples were also excluded from further microsatellite analysis. Nonetheless, in addition to the results based on those five neutral microsatellite markers, we also present results based 8 loci (excluding the uninformative CmA110 locus, see Supporting Information Appendix S2 for more information on the selection of loci) to increase molecular resolution since the primary purpose of the analyses was to identify hybrids and not to assess their population structure.

GenAlEx *vers*. 6.5 (Peakall & Smouse, 2006) was used to export the data in different input formats for downstream analyses. Genetic structure was assessed using STRUCTURE *vers*. 2.2.3 (Falush, Stephens, & Pritchard, 2003,2007; Hubisz, Falush, Stephens, & Pritchard, 2009; Pritchard, Stephens, & Donnelly, 2000) and DAPC plots in R *vers*. 2.12 (R Core Team, 2015). Together with the NewHybrids software (*vers*. 1.1 beta), we evaluated the assignment of specimens to one or the other parent species, or alternatively as putative first‐ or second‐generation hybrids (see Supporting Information Appendix S2 for more information on the generation and analyses of the microsatellite markers’ data).

## 3 RESULTS

### 3.1 Underwater visual surveys

**Figure 2:**
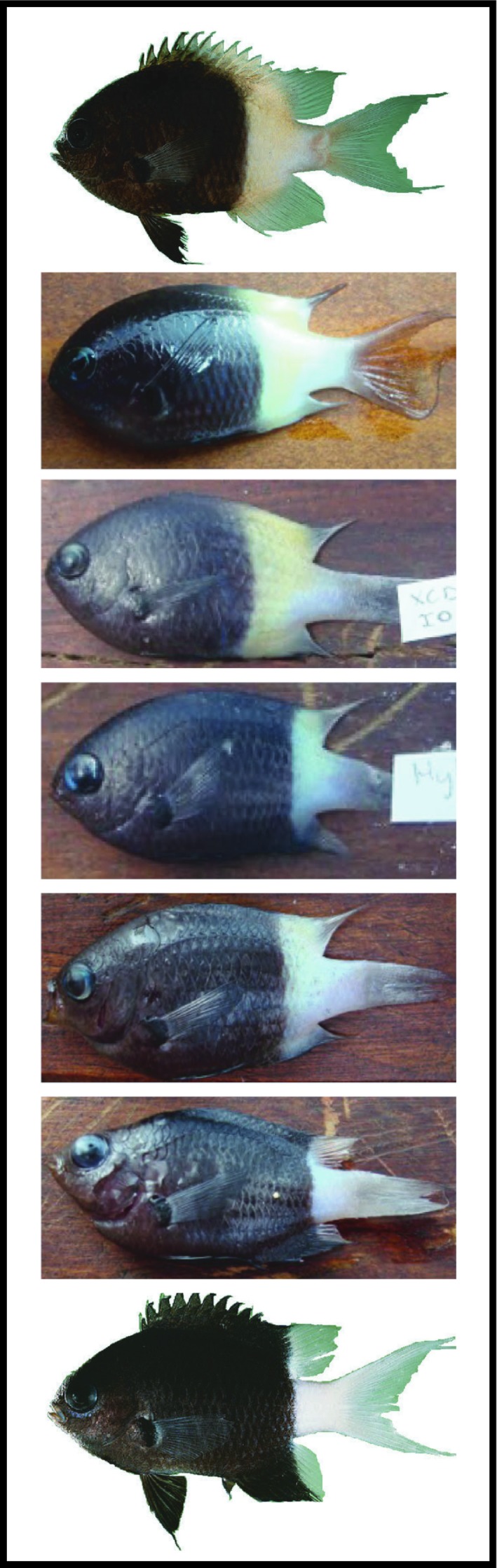
Lateral view of a tentative morphological gradient from *Chromis fieldi* (top) to *C. margaritifer* (bottom). The specimens, from top to bottom, are: *C. fieldi* (Mauritius, FishBase reference picture by J.E. Randall); XCD02 and XCD01 visually ID as *C. fieldi*; two putative hybrids (HYBRIDCHROMIS and COVEXCMDHY02); *C. margaritifer* (XCM22, Christmas Island, credit to J.P. Hobbs); and *C. margaritifer* (Marshall Islands, FishBase reference picture by J.E. Randall). The visual discrimination between purebred and hybrid specimens is mainly based on coloration (light yellowish vs. whiter caudal fin; and black vs. dark brown posterior body) and the position of the split/border line between light and dark colored body sections (rather “half and half” for *C. fieldi* vs. more restricted to the caudal fin only in *C. margaritifer*)

*Chromis fieldi*, *C. margaritifer*, and their hybrids were observed and collected at XMS, Australia, a recognized tropical marine suture zone among other reef fish species (Figure 1). Hybrids were identified by their intermediate coloration (Figure 2). Based on underwater visual survey data, *C. margaritifer* was approximately 50 times more abundant than *C. fieldi;* their hybrids were even rarer (total counts across all surveys: 475 *C. margaritifer*, 9 *C. fieldi*, and 1 hybrid). At COC, also within this suture zone, similar patterns in abundance were recorded for *C. margaritifer* and *C. fieldi* (Supporting Information Appendix S1). Although, at COC, no hybrids were observed at the time of this study's visual surveys, hybrids have been photographed at this location in the past.

At another recognized hybrid zone for reef fishes, SOC, *C. dimidiata* (restricted to the Red Sea), and intermediate colored individuals (putative hybrids with *C. fieldi*) were absent during our surveys. Here, only *C. fieldi* specimens were observed and collected.

### 3.2 Mitochondrial DNA

**Figure 3:**
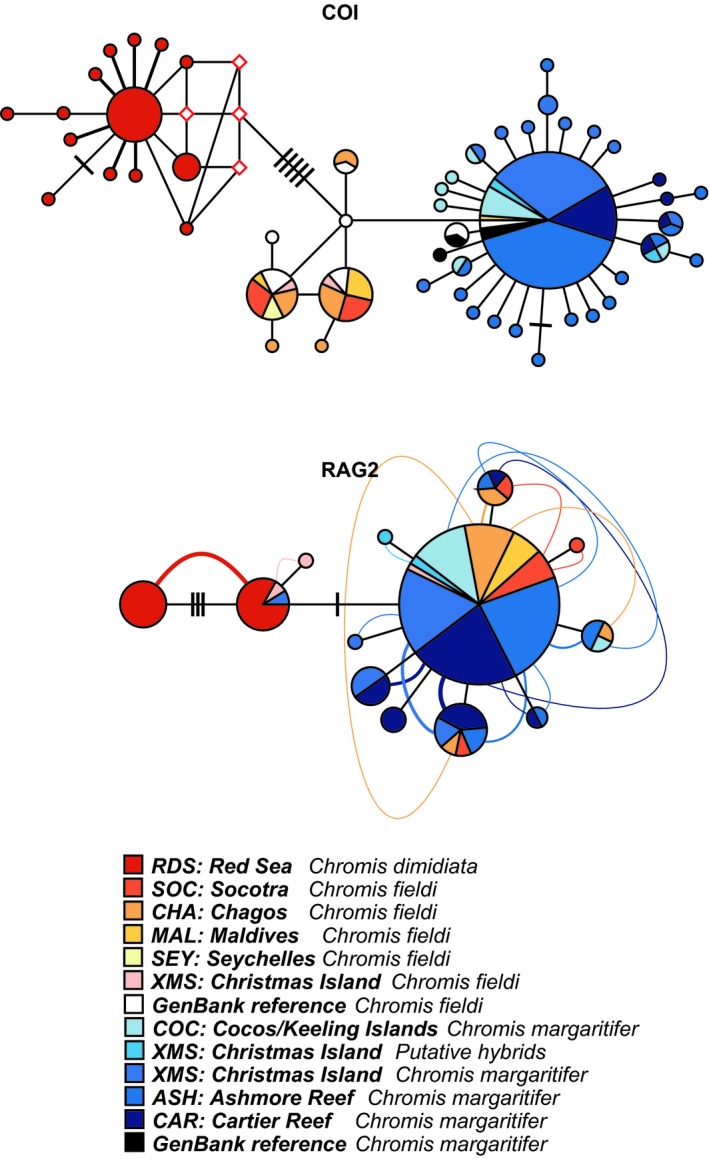
Phylogenetic relationship among *Chromis dimidiata*, *C. fieldi*, and *C. margaritifer* haplotypes represented in a median‐joining network of the mitochondrial cytochrome C oxidase subunit 1 (COI) and a network‐based haploweb (Flot, Couloux, & Tillier, 2010) of the nuclear recombination‐activating gene 2 (RAG2). Each circle represents a unique haplotype and circle sizes are proportional to its total frequency. The red rhombuses represent missing haplotypes. Each branch connecting different circles represents a single nucleotide change and black cross‐bars represent additional nucleotide changes. Curved lines connecting haplotypes indicate haplotypes occurring in heterozygous individuals. Colors denote sampled species and their geographical origin as indicated in the legend

Based on 578 bp of the COI gene, a total of 52 haplotypes were detected, with a haplotype diversity of 0.668. In the COI haplotype network, three distinct lineages of *Chromis* spp. were apparent, with all *C. dimidiata* haplotypes separated from *C. fieldi* haplotypes by at least six base pair substitutions (Figure 3). The majority (36 out of 39) of *C. fieldi* samples were separated from *C. margaritifer* by a single base pair substitution. The remaining three *C. fieldi* samples, RS7187 (Maldives), JF434879 (Reunion, GenBank voucher), and JF434880 (Madagascar, GenBank voucher) however grouped with the *C. margaritifer* lineage, despite those being morphotypically and biogeographically *C. fieldi*, which we interpret as evidence of introgression. The other two GenBank voucher sequences, JF434855 and JF434856 (Moorea, French Polynesia), morphotypically and genetically grouped with the *C. margaritifer* lineage (Figure 3). Both putative hybrids sampled from XMS grouped with the same *C. margaritifer* lineage (Figure 3).

Further, a low and negative Fu's Fs value (−13.44) was measured for the XMS population of *C. margaritifer*, and a relatively low *τ* value (1.23), which may be due to a recent population expansion of *C. margaritifer* at XMS compared to other sampling locations, or due to introgression (Table 1). In contrast, individuals sampled at Cocos/Keeling did not show such a trend, despite being within the suture zone (Table 1).

The K2P genetic distance between *C. dimidiata* and *C. margaritifer*/*C. fieldi* was 1.8%, whereas it was 0.3% between *C. margaritifer* and *C. fieldi*. To roughly estimate divergence time between *C. margaritifer* and *C. fieldi*, a molecular clock of 1% divergence per million years (MY) was applied (Bowen, Bass, Rocha, Grant, & Robertson, 2001; DiBattista et al., 2013). Divergence times between *C. margaritifer* and *C. fieldi* are approximately 0.3 MY, and 1.8 MY between *C. dimidiata* and *C. margaritifer*/*C. fieldi*; both dates corresponding to the Pleistocene (2.7 ~ 0.12 MYA).

### 3.3 Nuclear DNA

The 152 bp fragments of RAG2 from all *Chromis* samples contained 14 variable sites and 14 haplotypes, with a haplotype diversity of 0.478. For the network‐based haploweb, none of the *Chromis* lineages were distinct (Figure 3). There were four nucleotide substitutions between all *C. dimidiata* haplotypes. However, due to a high frequency of heterozygous individuals, all of the *C. dimidiata* haplotypes clustered as one lineage (Figure 3). While there was no distributional range overlap between *C. dimidiata* and populations of XMS *Chromis* spp., one *C. margaritifer* sample (ID: ETHELXCM14) and one *C. fieldi* sample (ID: COVEXCD02) from XMS shared an identical haplotype with *C. dimidiata* samples from the Red Sea (Figure 3), which we interpret as evidence of incomplete lineage sorting. Two nucleotide substitutions separated *C. dimidiata* samples from the rest of *C. ieldi* and *C. margaritifer* samples. No nucleotide changes differentiated *C. fieldi* from *C. margaritifer*. When taking heterozygous individuals into account, *C. fieldi* and *C. margaritifer* haplotypes were more closely related to each other (Figure 3).

### 3.4 Microsatellite analysis

**Figure 4:**
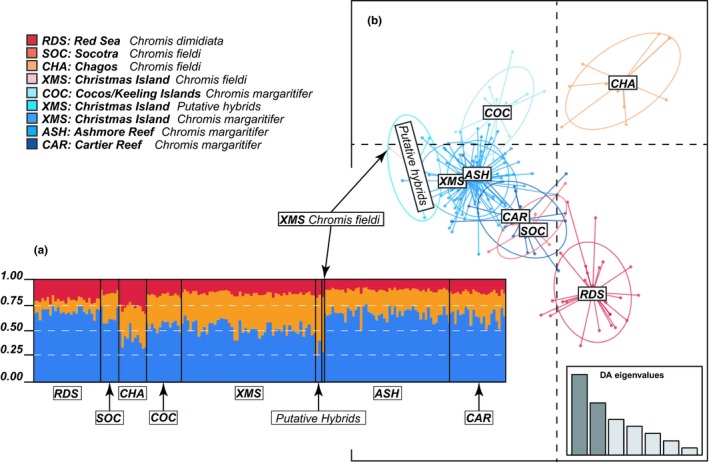
STRUCTURE bar plot (a) and DAPC scatterplot (b) of *Chromis dimidiata*, *C. fieldi*, and *C. margaritifer* based on five neutral microsatellite markers. Posterior probability of assignment of *Chromis* samples to one of two (*K* = 2) genotype clusters is shown in the bar plot (a), generated using a Bayesian clustering analysis of microsatellite genotypes. Dots in the DAPC scatterplot (b) represent individual genotypes, and identity categories for genotypes of each individual are indicated in the legend. Genetic variations within each population/species are represented by 95% inertia ellipses. Eigenvalue plots show the amount of genetic information retained by each successive function

Among our seven sampling locations, STRUCTURE analysis suggested no genetic differentiation between these three species (Figure 4a and Supporting Information Figure S1 in Appendix S2). The NewHybrids analysis also could not distinguish hybrids from purebred individuals due to apparent genetic similarities among the three species (Supporting Information Figure S2 in Appendix S2). However, the DAPC scatterplot did indicate a minor difference among *Chromis* spp. Three clusters corresponding to the three *Chromis* spp. considered in this study were visible in the scatterplot (Figure 4b and Supporting Information Figure S1 in Appendix S2). Individuals of *C. margaritifer* from ASH, CAR, XMS, and COC clustered together while *C. fieldi* from CHA and *C. dimidiata* from RDS clustered separately from the other two species. Putative hybrids sampled at XMS grouped with *C. margaritifer* from XMS. One of the *C. fieldi* samples (ID: COVEXCD01) collected at XMS was discarded due to repeated amplification failure despite multiple PCR attempts. The remaining *C. fieldi* sample (ID: COVEXCD02) from XMS was genetically more similar to *C. margaritifer* from XMS than other *C. fieldi* populations. (Figure 4b and Supporting Information Figure S1 in Appendix S2). Although samples from SOC were identified as *Chromis fieldi* morphologically, the DAPC suggested high genetic similarities to the *C. margaritifer* and *C. dimidiata* clusters. When using eight microsatellite markers (instead of the five), we observed similar genetic structuring patterns with NewHybrid (Supporting Information Figure S2 in Appendix S2), but the DAPC scatterplot showed clearer discrimination between the three *Chromis* spp. from different sampling locations, grouping according to their phenotypic species designation (Figure 4b and Supporting Information Figure S1 in Appendix S2) and the hybrids clustering with their population of origin: XMS.

## 4 DISCUSSION

We assessed putative hybridization between three tropical *Chromis* species: *C. dimidiata, C. fieldi,* and *C. margaritifer*. Importantly, while our study reports the first evidence of hybrids between *C. fieldi* and *C. margaritifer*, originating within the Cocos–Christmas suture zone and subsequently spreading their genes further to the western Indian Ocean, no hybrids or evidence for introgression was found within the Socotra suture zone between *C. dimidiata* and *C. fieldi*. In contrast to our hypothesis, the current biogeographical ranges of the latter two species also do not seem to overlap at Socotra, and *C. dimidiata* is likely limited to the Red Sea.

The approximate divergence time between *C. margaritifer* and *C. fieldi* also seems more recent (approx. 0.3 MYA) than that between *C. dimidiata* and *C. margaritifer*/*C. fieldi* (1.8 MYA). However, both estimates correspond to repeated glacial cycles of the Pleistocene Epoch (2.7 ~ 0.12 MYA). Indeed, Pleistocene ice ages played a key role in the formation of temporary barriers to larval dispersal, ultimately leading to speciation within many coral reef fish families. Both the Socotra and Cocos–Christmas suture zones are adjacent to shallow areas that represented putative barriers to gene flow during ice ages because these likely formed land bridges separating species on either side when sea levels dropped (DiBattista, Howard Choat, et al., 2016b; Rocha, Craig, & Bowen, 2007). These barriers are no longer there, which means dispersal of allopatric sister species (or recently diverging species) and potential interbreeding becomes possible at suture zones. Here, we find evidence for secondary contact between *C. margaritifer* and *C. fieldi* at the eastern Indian Ocean suture zone*,* with further introgression of *C. fieldi* to the Maldives. In contrast, at the western end of the chocolate‐dipped *Chromis* distributional range, environmental conditions in the Gulf of Aden and Arabian Sea may continue to prevent the Red Sea *C. dimidiata* from successfully dispersing out of the Red Sea and mixing with *C. fieldi* at the Socotra suture zone. Even though the Pleistocene land barrier at the Strait of Bab Al Mandab is now submerged, and some coral reef fishes are spreading beyond this historical barrier, dispersal of other Red Sea endemics may be hindered by strong environmental differences adjacent to this intermittently isolated body of water (DiBattista et al., 2013; DiBattista, Howard Choat, et al., 2016b). Thus, the two suture zones (Socotra vs. Cocos–Christmas) illustrate contrasting scenarios following divergence during Pleistocene ice ages. At Cocos–Christmas, secondary contact and hybridization is resulting in introgression between *C. fieldi* and *C. margaritifer*, while at Socotra present‐day environmental conditions may continue to isolate *C. dimidiata* from *C. fieldi*/*margaritifer,* or at least prevent successful settlement and survival of their larvae from adjacent seas.

Introgression at Cocos–Christmas is consistent with an active suture zone, where hybridization occurs among closely related species due to secondary contact between regional biotas from the Indian Ocean and Pacific Ocean (DiBattista, Whitney, et al., 2016a; Hewitt, 2000; Remington, 1968). In our assessment of putative hybridization among the three *Chromis* species, there was concordance between visual identification (phenotype) and genetic analysis of mitochondrial, nuclear, and to some extent microsatellite markers for most samples. The ability to detect incomplete lineage sorting among these lines of descent was limited by the low phylogenetic resolution of the RAG2 nuclear marker; but all markers displayed low genetic divergence between the three species and the presence of ongoing or historical introgression between *C. fieldi* and *C. margaritifer* was also supported with the remaining genetic markers, which was quite strong when using eight microsatellite markers as opposed to solely the five neutral microsatellites. Further, the genotypes of six individuals (COVEXCD01, COVEXCD02, HYBRIDCHROMIS, COVEXCMDHY02, ETHELXCM14, and RS7187; 2.6% of the samples) exhibited incongruence for their species assignment depending on the methodology applied. We interpret this incongruence as further evidence of hybridization.

Indeed, difficulties in distinguishing recently diverged species that are hybridizing are likely, given that some parts of the genome may show signs of gene flow, whereas other parts may not (Roux et al., 2016). Mitochondrial (e.g., COI), nuclear (e.g., RAG2), and microsatellites markers have different mutational rates and genealogies (Navajas & Boursot, 2003). According to our results, the mitochondrial COI and the nuclear microsatellites were capable of separating the three *Chromis* species despite ongoing gene flow (at least between *C. fieldi* and *C. margaritifer*), and the more microsatellite markers (eight vs. six loci) we included the clearer the segregation. The nuclear RAG2, however, has the slowest mutational rate among our genetic markers (Quenouille, Bermingham, & Planes, 2004) and displayed signatures of historical gene flow and incomplete lineage sorting (specifically between *C. dimidiata* and *C. fieldi*/*margaritifer*) but no evidence of more recent divergence between species (mainly, *C. fieldi*/*margaritifer*). By combining results from these three complimentary markers, along with biogeographical and phenotypic information, we were able to posit testable hypotheses for inconsistent assignment of the six aforementioned samples:

1. The *C. fieldi* collected from Christmas Island (COVEXCD01) had a typical *C. fieldi* mitochondrial COI haplotype but its nuclear microsatellite loci grouped with *C. margaritifer*. This mismatch strongly suggests ongoing hybridization at Christmas Island, where the distributions of these two Chromis species overlap. This sample is probably an F1 hybrid, or a backcrossed individual, meaning the offspring of a male hybrid parent and a female *C. fieldi*. Due to the fourfold, slower evolutionary rate of the nuclear RAG2 compared to COI, there was no separation between *C. margaritifer* and *C. fieldi* at this marker.
2. In the case of COVEXCD02, the similarity of its RAG2 haplotype with those of *C. dimidiata* from the Red Sea suggests incomplete lineage sorting among *C. fieldi *and *C. dimidiata*, despite the substantial geographic separation between these sites (RDS and XMS, ~7,300 km).
3. Similar to (2), evidence of incomplete lineage sorting was detected at Christmas Island based on the *C. margaritifer* ETHELXCM14.
4. The mismatch between phenotype and mitochondrial COI haplotype for the Maldivian *C. fieldi* (RS7187) indicates putative introgression caused by hybridization between the two species, *C. margaritifer* and *C. fieldi*. This hypothesis of introgression is further supported by previously collected *C. fieldi *samples from Reunion (JF434880) and Madagascar (JF434879), which also carried a *C. margaritifer* COI haplotype. Hence, these putative hybrids or backcrossed individuals likely dispersed from Christmas Island and introgressed with *C. fieldi* at the Maldives. Furthermore, *C. margaritifer* COI haplotypes appear to have introgressed or backcrossed into *C. fieldi* populations at the western edge of their range (i.e., western Indian Ocean). This pattern of introgression in the eastern Indian Ocean (XMS/COC) and a westward pattern of dispersal and introgression has already been reported among other hybridizing reef fishes (DiBattista, Whitney, et al., 2016a; Marie et al., 2007). If this holds true, hybridization of *C. fieldi* in a restricted area, at the eastern edge of its range, could have far greater “downstream” implications because it would also lead to introgression across the rest of its biogeographical range.
5. Lastly, the two putative hybrids collected at Christmas Island, (HYBRIDCHROMIS, and COVEXCMDHY02) could not be genetically confirmed with the markers used in this study (only RAG2 provided genetic evidence). This, however, is not necessarily evidence against hybridization. The detection of an intermediate colour morph has proven itself a reliable indicator for hybridization of several coral reef fish taxa (DiBattista et al., 2015). Thus, together with the genetic confirmation of hybridization among other samples in this study, we suggest that these last two putative hybrids may represent backcrossed offspring from hybrid parents despite ambiguous genetic results.

Evidence of introgression and the confirmation of hybridization between closely related reef fish species highlights the importance of suture zones as natural evolutionary laboratories. Indeed, even though many reef fish species are widely distributed, it is the interactions at edges of their range that have a disproportionally large effect on a species’ genotype (Budd & Pandolfi, 2010). Alongside other cases of hybridization at the Christmas–Cocos Islands (Hobbs et al., 2009; Montanari, Herwerden, Pratchett, Hobbs, & Fugedi, 2012; Payet et al., 2016), our findings further support this Indo‐West Pacific suture zone as a hybridization hotspot. Moreover, it highlights different outcomes at two putative suture zones (Socotra vs. Cocos–Christmas), which demonstrate how past (geological) and current (ecological) processes can drive evolution of coral reef fishes.

## CONFLICT OF INTEREST

The authors declare no competing interests.

## AUTHOR CONTRIBUTIONS

J.D.D., J‐P.A.H., and S.H. conceived the idea for this study. J.D.D., J‐P.A.H., and M.J.T. collected tissue samples; J‐P.A.H. conducted abundance surveys; S.H. and J.D.D. produced DNA sequences; S.H., J.D.D., J‐P.A.H., V.R.S., and D.L.C. analyzed the data. S.H. and V.R.S. interpreted and discussed the results leading to the final construction of the manuscript. J.D.D., J‐P.A.H., V.R.S., M.J.T., and M.L.B. also made the significant contributions to the manuscript.

## Data Availability

DNA sequences can be found in Genbank, accession numbers: MH287769–MH287999. Any other missing information can be supplied per email upon request to the corresponding author.
