# Supplementary material for: An examination of introgression and incomplete lineage sorting among three closely related species of chocolate‐dipped damselfish (genus: Chromis)
Source: Ecol Evol. 2019 Apr 19;9(9):5468–78. doi: 10.1002/ece3.5142 (PMC6509375; doi:10.1002/ece3.5142)
Supplement: Supplementary file 1 [file ECE3-9-5468-s001.docx]

*Journal of Biogeography*

**SUPPORTING INFORMATION**

**An examination of introgression and incomplete lineage sorting among three closely related species of chocolate-dipped damselfish (genus: Chromis)**

Song He, Vanessa Robitzch, Jean-Paul A. Hobbs, Michael J. Travers, Diego Lozano-Cortés, Michael L. Berumen, and Joseph D. DiBattista

**APPENDIX S1: Methods for sequence analyses**

jModelTest *vers.* 1.0.1 (Guindon & Gascuel, 2003) was used with an Akaike information criterion (*AIC*) test to determine the best nucleotide substitution model in each species for the mtDNA dataset; the TrN (Tamura & Nei, 1993) model was selected in all cases. Genetic distances between different lineages were calculated by MEGA6 using the Kimura 2-parameter (K2P) model with default settings (Tamura et al., 2013). ARLEQUIN *vers.* 3.1 (Excoffier et al., 2005) was used to calculate haplotype (*h*) and nucleotide diversity (*π*), as well as to test for range-wide patterns of population structure in *Chromis fieldi* and *C. margaritifer* (Table 1). Population pairwise *Φ_ST_* statistics were generated in ARLEQUIN to identify particular sites associated with genetic partitioning. Significance was tested by permutations (*N* = 10,000) and *P*-values adjusted by the modified false discovery rate method (Table 1, as per Narum, 2006).

Deviations from selective sequence neutrality were assessed by calculating Fu’s *Fs* (Fu, 1997) for each population using ARLEQUIN; significance was tested with 10,000 permutations. Each site was also fitted with the population parameter *τ* (Rogers & Harpending, 1992), but given the uncertainty in generation time and mutation rates for these species, and in some cases small sample sizes, we limited our inferences to rank order comparisons of this parameter between regions.

Allelic status of nuclear sequences trimmed to a uniform length with more than one heterozygous site was estimated using the Bayesian program PHASE *vers.* 2.1 (Stephens & Donnelly, 2003) as implemented in the software DnaSP *vers.* 5.0 (Librado & Rozas, 2009). We conducted three runs in PHASE for each dataset with a burn-in of 10,000 and 200,000 iterations. All runs returned consistent allele identities and PHASE was able to differentiate most alleles with >95% probability except for 2 alleles of *C. dimidiata*, 5 alleles of *C. fieldi*, and 7 alleles of *C. margaritifer*, which were all excluded from further nuclear DNA analysis.

Evolutionary relationships among COI haplotypes and RAG2 alleles within and between each species were assessed with a median-joining network constructed with the program NETWORK *vers.* 4.5.1.0 ([www.fluxus-engineering.com/network_terms.htm](http://www.fluxus-engineering.com/network_terms.htm)) using default settings (Bandelt, 1999). The RAG2 haploweb (Flot et al., 2010) was derived from median-joining networks by drawing curves connecting haplotypes that co-occur in heterozygous individuals.

**REFERENCES**

Bandelt, H.-J., Peter Forster, Arne Röhl. (1999). Median-joining networks for inferring intraspecific phylogenies. *Molecular Biology and Evolution*, **16**, 37-48.

Excoffier, L., Laval, G. & Schneider, S. (2005). Arlequin (version 3.0): an integrated software package for population genetics data analysis. *Evolutionary Bioinformatics Online*, **1**, 47-50.

Flot, J.-F., Couloux, A. & Tillier, S. (2010). Haplowebs as a graphical tool for delimiting species: a revival of Doyle's" field for recombination" approach and its application to the coral genus *Pocillopora* in Clipperton. *BMC Evolutionary Biology*, **10**, 372-386.

Fu, Y.-X. (1997). Statistical tests of neutrality of mutations against population growth, hitchhiking and background selection. *Genetics*, **147**, 915-925.

Guindon, S. & Gascuel, O. (2003). A simple, fast, and accurate algorithm to estimate large phylogenies by maximum likelihood. *Systematic biology*, **52**, 696-704.

Librado, P. & Rozas, J. (2009). DnaSP v5: a software for comprehensive analysis of DNA polymorphism data. *Bioinformatics*, **25**, 1451-1452.

Narum, S.R. (2006). Beyond Bonferroni: less conservative analyses for conservation genetics. *Conservation Genetics*, **7**, 783-787.

Rogers, A.R. & Harpending, H. (1992). Population growth makes waves in the distribution of pairwise genetic differences. *Molecular Biology and Evolution*, **9**, 552-569.

Stephens, M. & Donnelly, P. (2003) A comparison of bayesian methods for haplotype reconstruction from population genotype data. *The American Journal of Human Genetics*, **73**, 1162-1169.

Tamura, K. & Nei, M. (1993). Estimation of the number of nucleotide substitutions in the control region of mitochondrial DNA in humans and chimpanzees. *Molecular Biology and Evolution*, **10**, 512-526.

Tamura, K., Stecher, G., Peterson, D., Filipski, A. & Kumar, S. (2013). MEGA6: molecular evolutionary genetics analysis version 6.0. *Molecular Biology and Evolution*, **30**, 2725-2729.
