# Supplementary material for: An examination of introgression and incomplete lineage sorting among three closely related species of chocolate‐dipped damselfish (genus: Chromis)
Source: Ecol Evol. 2019 Apr 19;9(9):5468–78. doi: 10.1002/ece3.5142 (PMC6509375; doi:10.1002/ece3.5142)
Supplement: Supplementary file 2 [file ECE3-9-5468-s002.docx]

*Journal of Biogeography*

**SUPPORTING INFORMATION**

**An examination of introgression and incomplete lineage sorting among three closely related species of chocolate-dipped damselfish (genus: Chromis)**

Song He, Vanessa Robitzch, Jean-Paul A. Hobbs, Michael J. Travers, Diego Lozano-Cortés, Michael L. Berumen, and Joseph D. DiBattista

**APPENDIX S2: Methods for microsatellite marker analyses**

Genetic diversity metrics, including number of alleles (*N_a_*) and observed (*H_O_*) and expected heterozygosities (*H_E_*), were calculated in GenePop *vers.* 4.3 (Rousset, 2008). Null alleles, probability of departure from Hardy-Weinberg equilibrium (HW), and linkage disequilibrium (LD) were estimated using GenePop. Thereafter, CmA115 was further excluded from analysis due to > 5% null alleles in most populations. All five remaining microsatellite loci passed the aforementioned tests.

Population pairwise *F_ST_* statistics were generated in ARLEQUIN to identify particular sites associated with genetic partitioning. Significance was tested by permutations (*N* = 10,000) and *P*-values adjusted by the modified false discovery rate method (Narum, 2006). STRUCTURE *vers.* 2.2.3 (Pritchard et al., 2000; Falush et al., 2003, 2007; Hubisz et al., 2009) was used to assess genetic structure based on Bayesian multi-locus clustering between the different *Chromis* species. STRUCTURE runs used 1,000,000 MCMC and a burn-in period of 100,000, with 10 runs per *K* for K = 1 to K = 10 (representative of the total number of sampling locations plus three putative hybrid individuals). The most likely number of genetic clusters was calculated with STRUCTURE HARVESTER and the Evanno Method (Earl & VonHoldt, 2012). This method can only differentiate the most likely number of K for K > 1. Thus, it is not possible to detect a K=1 as most likely. We therefore used the graphical barplot representation of the final STRUCTURE output to interpret results and present the results based on *K* = 3 for five and eight loci (to visualise putative hybrids). Additionally, a model-based Bayesian clustering method implemented in NEWHYBRIDS (Anderson & Thompson, 2002) was utilized to distinguish hybrids from pure breeding classes. One loci (CmA110) was removed from the data set due to lack of variation among all of the samples; only samples with data for at least six of the remaining eight loci were used in the analysis to minimize the effect of missing data. Hence, our data set was composed of 184 individuals with on average 5.2% missing data across eight loci. Six possible genotype frequency classes (purebred individuals, F1 and F2 hybrids, and backcrossed individuals of both species) were considered and were specified in terms of the expected proportion of loci originating from one or the other species (following Anderson & Thompson, 2002). Individuals with less than 90% probability of species assignment were considered hybrids of mixed ancestry. To avoid influencing the assignment of samples, no prior information regarding the status or class of individuals was used. The analysis in NEWHYBRIDS was performed over a single run using Jeffrey’s prior for both theta (allele frequencies) and pi (mixing proportion) with a burn-in of 5,000 MCMC sweeps. The posterior probability of each individual belonging to each class was calculated from Monte Carlo averages following more than 20,000 sweeps.

To further investigate population genetic structure among these three species, discriminant analysis of principal components (DAPC) (Jombart et al., 2010) was performed using the five microsatellite loci (Cm_A119, Cm_B117, Cm_D006, Cm_A011, and Cm_B102). This method is designed to assign individuals to predefined groups based on their genetic information (Jombart et al., 2010). DAPC was implemented in R *vers.* 2.12 (R Core Team, 2015) using the functions *dudi.pca* and *dapc* from the packages ade4, adegenet, and MASS. Results were visualised in a scatterplot using adegenet (Jombart, 2008; Jombart et al., 2010).


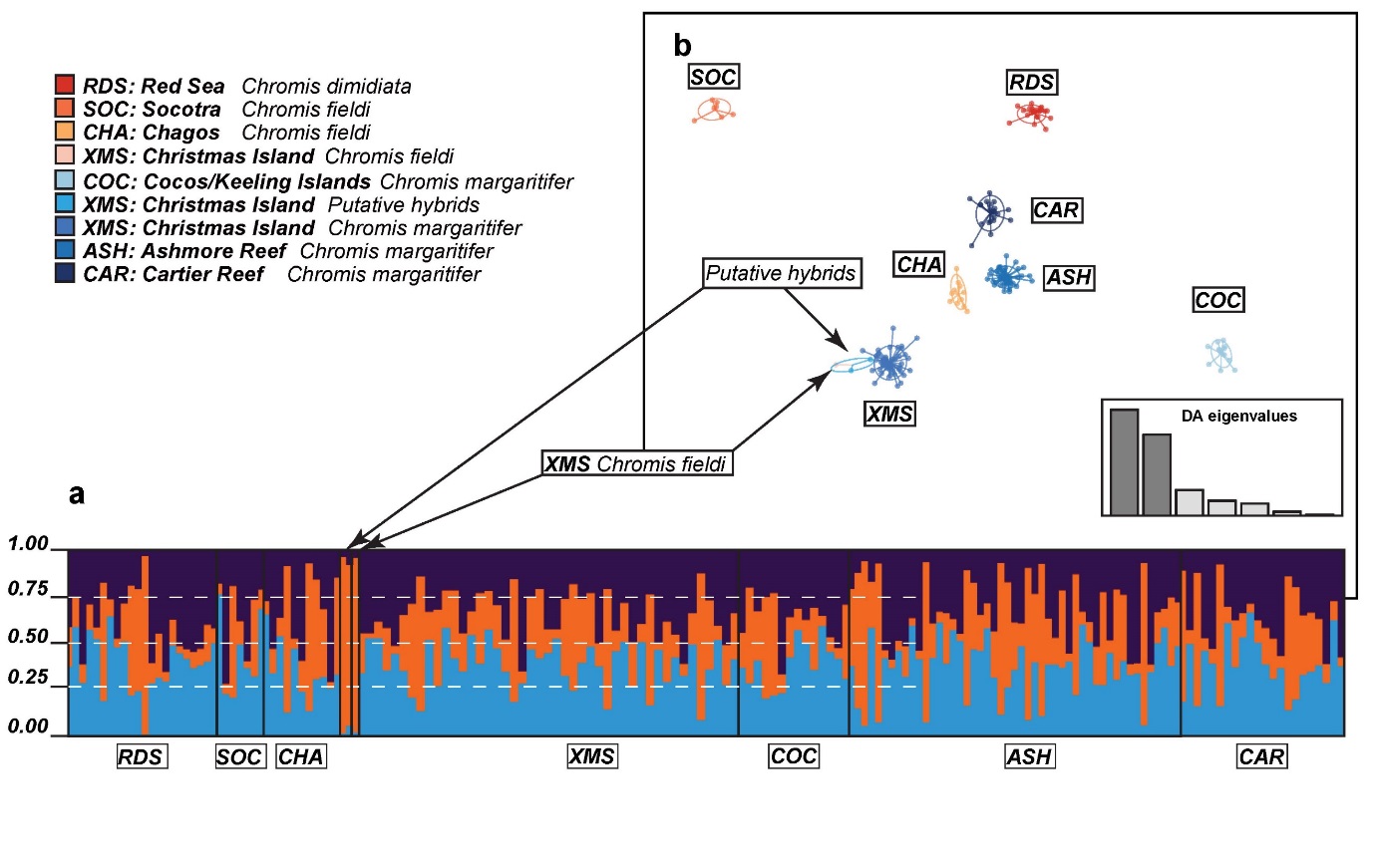


**Appendix S2 Figure S1:** STRUCTURE bar plot (a) and DAPC scatter plot (b) of *Chromis dimidiata*, *C. fieldi*, and *C. margaritifer* based on eight microsatellite markers. Posterior probability of assignment of *Chromis* samples to one of two (*K* = 3) genotype clusters are shown in the bar plot (a), generated using a Bayesian clustering analysis of 8 loci microsatellite genotypes. Dots in the DAPC scatter plot (b) represent individual genotypes, and identity categories for genotypes of each individual are indicated in the legend. Genetic variations within each population/species are represented by 95% inertia ellipses. Eigenvalue plots show the amount of genetic information retained by each successive function.


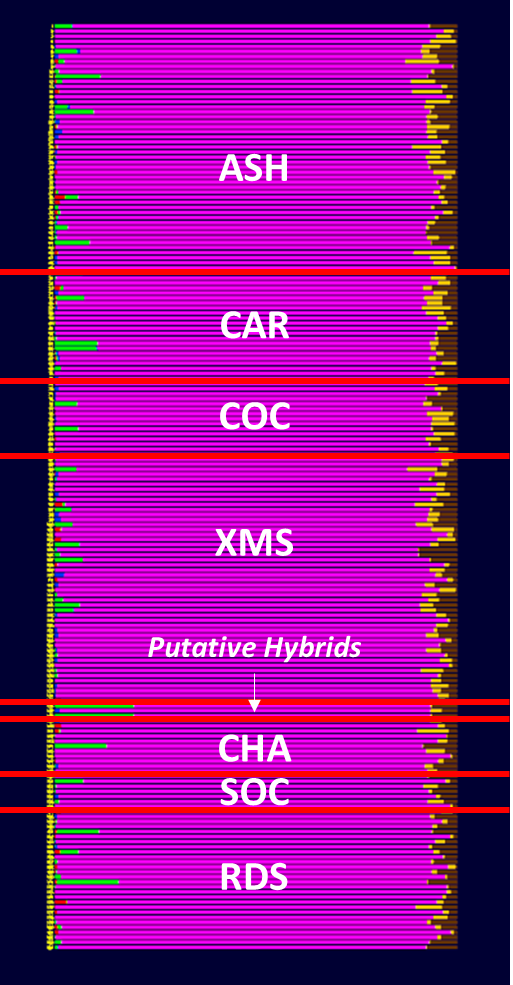


**Appendix S2 Figure S2:** The posterior probability of assignment of sampled *Chromis* spp. genotype classes following a Bayesian clustering analysis of 5 loci microsatellite genotypes in NEWHYBRIDS. Due to the apparent genetic similarities among these three species, hybrids and purebred individuals could not be resolved during this analysis.

**REFERENCES**

Anderson, E.C. & Thompson, E.A. (2002). A model-based method for identifying species hybrids using multilocos genetic data. *Genetics*, **160**, 1217-1229.

Earl, D.A. & VonHoldt, B.M. (2012). STRUCTURE HARVESTER: a website and program for visualizing STRUCTURE output and implementing the Evanno method. *Conservation genetics resources*, **4**, 359-361.

Falush, D., Stephens, M. & Pritchard, J.K. (2003). Inference of population structure using multilocus genotype data: linked loci and correlated allele frequencies. *Genetics*, **164**, 1567-1587.

Falush, D., Stephens, M. & Pritchard, J.K. (2007). Inference of population structure using multilocus genotype data: dominant markers and null alleles. *Molecular Ecology Resources*, **7**, 574-578.

Hubisz, M.J., Falush, D., Stephens, M. & Pritchard, J.K. (2009). Inferring weak population structure with the assistance of sample group information. *Molecular Ecology Resources*, **9**, 1322-1332.

Jombart, T. (2008). adegenet: a R package for the multivariate analysis of genetic markers. *Bioinformatics*, **24**, 1403-1405.

Jombart, T., Devillard, S. & Balloux, F. (2010). Discriminant analysis of principal components: a new method for the analysis of genetically structured populations. *BMC Genetics*, **11**, 94-109.

Narum, S.R. (2006). Beyond Bonferroni: less conservative analyses for conservation genetics. *Conservation Genetics*, **7**, 783-787.

Pritchard, J.K., Stephens, M. & Donnelly, P. (2000). Inference of population structure using multilocus genotype data. *Genetics*, **155**, 945-959.

R Core Team. (2015). R: A language and environment for statistical computing. R Foundation for Statistical Computing, Vienna, Austria. *URL* [*http://www.R-project.org/*](http://www.R-project.org/)*.*

Rousset, F. (2008). Genepop 007: a complete re‐implementation of the genepop software for Windows and Linux. *Molecular Ecology Resources*, **8**, 103-106.
